# Get the Balance Right: ROS Homeostasis and Redox Signalling in Fruit

**DOI:** 10.3389/fpls.2019.01091

**Published:** 2019-09-18

**Authors:** Guillaume Decros, Pierre Baldet, Bertrand Beauvoit, Rebecca Stevens, Amélie Flandin, Sophie Colombié, Yves Gibon, Pierre Pétriacq

**Affiliations:** ^1^UMR 1332 BFP, INRA, Univ. Bordeaux, Villenave d’Ornon, France; ^2^UR-1052 GAFL, INRA, CS60094, Montfavet, France; ^3^MetaboHUB-Bordeaux, MetaboHUB, Phenome-Emphasis, Villenave d’Ornon, France

**Keywords:** redox, fruit, ROS, metabolism, NAD, glutathione, tomato, ascorbate

## Abstract

Plant central metabolism generates reactive oxygen species (ROS), which are key regulators that mediate signalling pathways involved in developmental processes and plant responses to environmental fluctuations. These highly reactive metabolites can lead to cellular damage when the reduction-oxidation (redox) homeostasis becomes unbalanced. Whilst decades of research have studied redox homeostasis in leaves, fundamental knowledge in fruit biology is still fragmentary. This is even more surprising when considering the natural profusion of fruit antioxidants that can process ROS and benefit human health. In this review, we explore redox biology in fruit and provide an overview of fruit antioxidants with recent examples. We further examine the central role of the redox hub in signalling during development and stress, with particular emphasis on ascorbate, also referred to as vitamin C. Progress in understanding the molecular mechanisms involved in the redox regulations that are linked to central metabolism and stress pathways will help to define novel strategies for optimising fruit nutritional quality, fruit production and storage.

## Introduction

Reduction-oxidation (redox) processes are a major consequence of the presence of ground-state oxygen gas (O_2_, constituting c.a. 20.8% of the atmosphere) as a natural oxidant on Earth. Photosynthetic organisms (e.g. cyanobacteria, green algae, plants) produced O_2_ by the light-driven splitting of water (H_2_O) during oxygenic photosynthesis (Foyer, 2018). In other words, photosynthesis functionally houses redox reactions in plants that are underpinned by the transfer of electrons between a donor and an acceptor. Consequently, this redox biochemistry generates the so-called reactive oxygen species (ROS). In tissues with low or no photosynthesis, such as roots and fruits, mitochondria can also drive the flow of electrons, thereby generating energy and ROS (Schertl and Braun, 2014).

**Figure 1:**
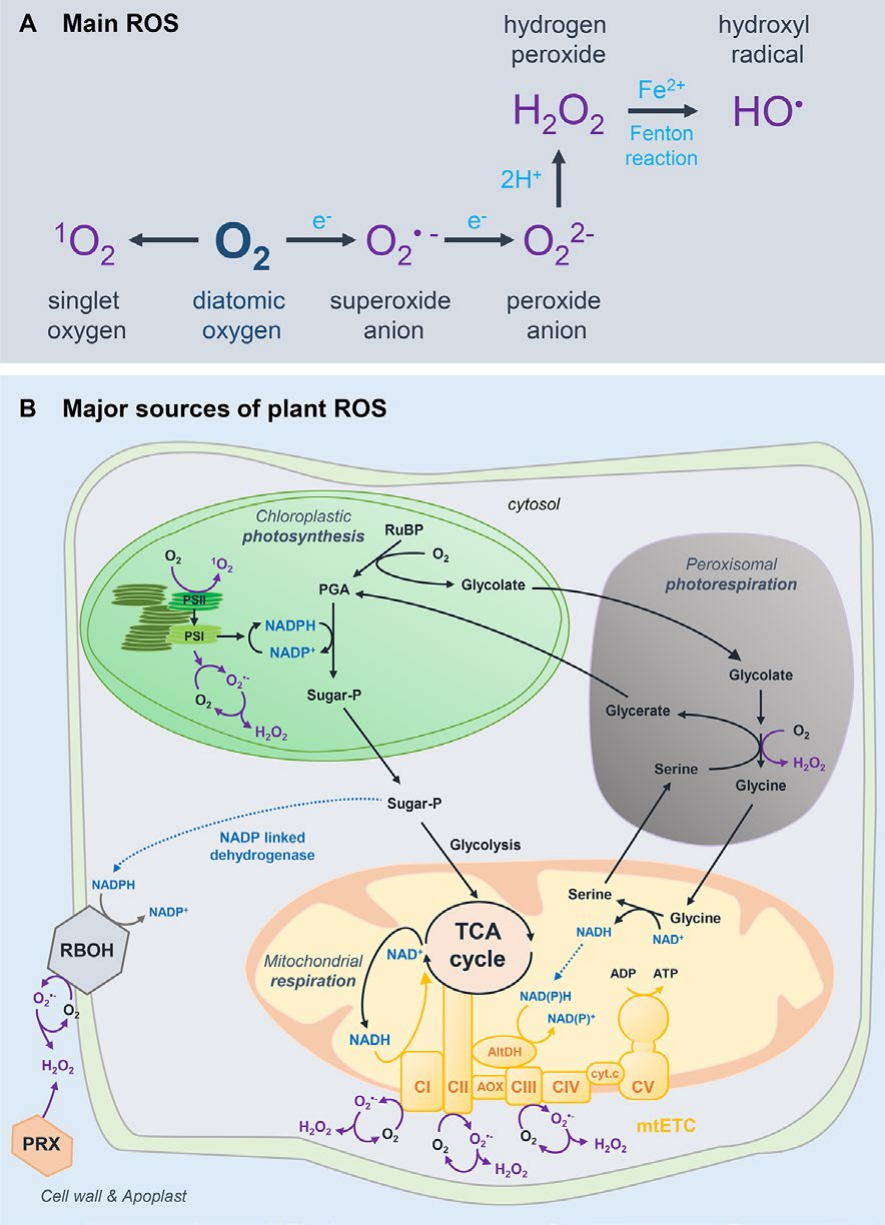
Basics of ROS biology in plants. **(A)** Main reactive oxygen species (ROS) formed from atmospheric oxygen (O_2_). **(B)** Major sources of ROS in plant cells. Excited triplet chlorophylls (Chl) can exacerbate the formation of singlet oxygen, mostly by the photosystem (PS) II reaction centre by photodynamic activation of ground-state oxygen (Fischer et al., 2013). In addition to singlet oxygen, superoxide and hydrogen peroxide can be formed in the chloroplast through the reduction of molecular oxygen, especially by the PSI. The four main complexes (CI–CIV) of the mitochondrial electron transport chain (mtETC) and the ATP synthase (CV) are represented in yellow. Among these complexes, CI (EC 1.6.5.3, NADH: ubiquinone oxidoreductase), CII (EC 1.3.5.1, succinate dehydrogenase) and CIII (EC 1.10.2.2, ubiquinol: cytochrome C oxidoreductase) are ROS-generating systems (Quinlan et al., 2012). mtETC, mitochondrial electron transfer chain; PRX, peroxidase; TCA, tricarboxylic acid; CI-V, complex I-V; AOX, alternative oxidase; AltDH, alternative dehydrogenase; cyt. c, cytochrome c; RuBP, ribulose 1,5-bisphosphate; PGA, phosphoglycerate; Sugar-P, sugar-phosphate; PS, photosystem.

Reactive oxygen species encompass highly reactive molecules that are partially reduced or excited forms of O_2_ including singlet oxygen (^1^O_2_), hydrogen peroxide (H_2_O_2_), the superoxide anion (O_2_^•−^) and the hydroxyl radical (OH•) (Apel and Hirt, 2004) (**Figure 1A**). Decades of research on redox biology pointed to a dual role for ROS both as toxic by-products of aerobic metabolism and as powerful signals that modulate plant functions (Mittler et al., 2011; Mittler, 2017; Foyer, 2018). With respect to this ambivalent concept, several ROS (e.g. H_2_O_2_) are produced during plant metabolism and development and in response to a fluctuating environment.

Fruits, including fleshy fruits, are peculiar plant organs of great economic importance (e.g. 866 Mt worldwide in 2016, www.fao.org/faostat). They constitute a remarkable source of food worldwide and contain a plethora of natural compounds with various benefits for human health and nutrition, including vitamins, nutrients, fibres, proteins and minerals (Baldet et al., 2014; Rodriguez-Casado, 2016; Padayachee et al., 2017). Despite having high concentrations in carbohydrates, fruits usually exhibit reduced photosynthetic activity, but sometimes high respiration rates, in particular for climacteric fruits, such as tomato (Roch et al., 2019). As for other vegetative plant tissues, fruit biology involves redox reactions and generates ROS. Some fruits are major sources of antioxidants, such as ascorbate, which scavenge ROS (Gest et al., 2013b; Smirnoff, 2018).

To date, there is no global overview of the involvement of oxidative metabolism in fruit biology, despite some fairly recent reviews on ripening and photo-oxidative stress (Tian et al., 2013; Osorio et al., 2013a; Cocaliadis et al., 2014; Muñoz and Munné-Bosch, 2018). This present review aims at updating our current knowledge on redox biology of fleshy fruits. We provide an overview of the profusion of natural compounds having antioxidant properties and examine the importance of redox regulation in plant metabolism for development and stress responses. We also discuss the relevance of metabolic modelling for the study of redox fluxes in plants, which should help to improve knowledge on the link between metabolism and cell redox status and therefore to evaluate strategies for optimal fruit production and storage.

## The Basics of Redox Biology in Plant Cells

For decades, redox signalling has been perceived as a balance between low levels of ROS acting as signals to trigger signalling cascades that adjust plant functions and high levels of ROS causing oxidative cellular damage (Apel and Hirt, 2004). Currently, the paradigm of redox biology tends to display a bigger and clearer picture of the redox network, especially in plants where multiple sources of ROS are possible and associated with many ‘ROS-processing systems’ (Noctor et al., 2018). Spatial, temporal, metabolic and antioxidant specificities are multiple factors that can influence redox signalling. Whilst redox biology in fruit is clearly fragmentary, the concepts that originate from foliar tissues are useful whilst waiting for comprehensive studies that bring more substantial levels of knowledge. This section briefly describes the major sources of ROS that are found in plant cells and the systems that process them.

### ROS Formation in Plants

The three main sources of plant ROS are the chloroplastic photosynthesis, the mitochondrial respiration and the peroxisomal photorespiration cycle (**Figure 1B**). The photosynthetic transport chain is assumed to be the major source of plant ROS in photosynthetic tissues. Superoxide can directly exert its signalling function or be chemically reduced or dismutated to H_2_O_2_. Dismutation of H_2_O_2_ can be accelerated by superoxide dismutases (SODs; EC 1.15.1.1), which are pivotal in regulating the redox status of the plant cell (Smirnoff and Arnaud, 2019). Importantly, H_2_O_2_ is more likely to trigger transduction signals over longer cellular distances (e.g. into the nucleus) as it has a longer lifespan, a greater diffusion distance and stability as compared to ^1^O_2_ (Exposito-Rodriguez et al., 2017; Mittler, 2017).

The photorespiratory cycle makes photosynthesis possible by scavenging 2-phosphoglycolate, which is toxic for the cell (Hodges et al., 2016). This highly compartmentalised pathway involving the chloroplast, peroxisome and mitochondrion is critical in generating H_2_O_2_ through the activity of peroxisomal glycolate oxidase (EC 1.1.3.15). Of course, the contribution of peroxisomal volume to total cell volume is small: 1% for peroxisomes compared to 12% for chloroplasts in leaves (Queval et al., 2011). Nonetheless, peroxisomes are predicted to be a major source of hydrogen peroxide in active photorespiratory cells. Furthermore, photorespiration-driven H_2_O_2_ is solely dismutated by peroxisomal catalase, which is commonly used as a redox marker of the peroxisome (Smirnoff and Arnaud, 2019, 202). In fruit, a high activity of the ascorbate recycling enzyme monodehydroascorbate reductase was observed in tomato fruit peroxisomes (Gest et al., 2013a), which supports the idea of an important role for peroxisomes in fruit redox homeostasis.

In nonphotosynthetic tissues, energy mostly originates from mitochondrial activity, which also contributes to generate ROS (Quinlan et al., 2012) (**Figure 1B**). The tricarboxylic acid cycle reduces NAD^+^ into NADH in the mitochondrion, which is fundamental to ensure that cellular respiration produces ATP *via* oxidative phosphorylation (Millar et al., 2011) (**Figure 1B**). Thus, mitochondria are tightly linked to NAD(H) turnover (Gakière et al., 2018a). As for the chloroplast, specific SODs dismutate rapidly O_2_^•−^ into H_2_O_2_ (Smirnoff and Arnaud, 2019). Besides ROS-generating systems, plant mitochondria specifically harbour alternative NADP(H) dehydrogenases that face both the matrix and the intermembrane space, as well as alternative oxidase (AOX) (**Figure 1B**). These enzymes are alternative respiratory routes, which do not produce energy, but allow viability when the enzymes of the main pathway are affected (Rasmusson et al., 2008, Rasmusson et al., 2009; Schertl and Braun, 2014). Alternative NADP(H) dehydrogenases can remove excess of reducing power in the mitochondria, which will balance the redox poise.

In addition, plant ROS can originate from other ROS-generating systems, including NADPH and xanthine oxidases. The NADPH oxidases (EC 1.6.3.1) are well-studied key players in ROS production (**Figure 1B**), most particularly with respect to biotic and abiotic environmental stresses (Torres and Dangl, 2005; Suzuki et al., 2011; Mittler, 2017). Xanthine dehydrogenases (EC 1.17.1.4, XDH) are important enzymes involved in the hydroxylation of hypoxanthine to xanthine, but can also form O_2_^•−^ when molecular oxygen is used as the electron acceptor. Whilst XDHs in mammals can be converted into xanthine oxidases that produce both O_2_^•−^ and H_2_O_2_, plant XDHs only form O_2_^•−^, which can be swiftly dismutated into H_2_O_2_ (Yesbergenova et al., 2005; Ma et al., 2016). In complement, class III peroxidases (PXs; EC 1.11.1.7) are heme-containing enzymes that produce O_2_^•−^ and H_2_O_2_ at the apoplast (Bindschedler et al., 2006; Cosio and Dunand, 2009; Daudi et al., 2012), although H_2_O_2_ formation is favoured at high pH in the presence of reductants (O’Brien et al., 2012). Peroxidases are also able to oxidise a donor and thereby process H_2_O_2_ (Lüthje and Martinez-Cortes, 2018).

For fruit tissues, however, knowledge is still lacking on the exact contribution of each source of ROS. Of course, due to low photosynthetic metabolism in fruit, one could predict different contributions than for leaves, which further depends on the plant species that exhibit diverse biochemical pathways able to scavenge and process cellular ROS. Even though mitochondria, peroxisomes and the apoplast are assumed to be leaders in ROS production in flowers and fruits (Qin et al., 2009a, Qin et al., 2009b; Rogers and Munné-Bosch, 2016), further research on fruit ROS is necessary to unveil the actual ROS-generating compartments and processes that mostly contribute to ROS production in fruit tissues.

### Systems for ROS Scavenging and Processing in Plants

**Figure 2:**
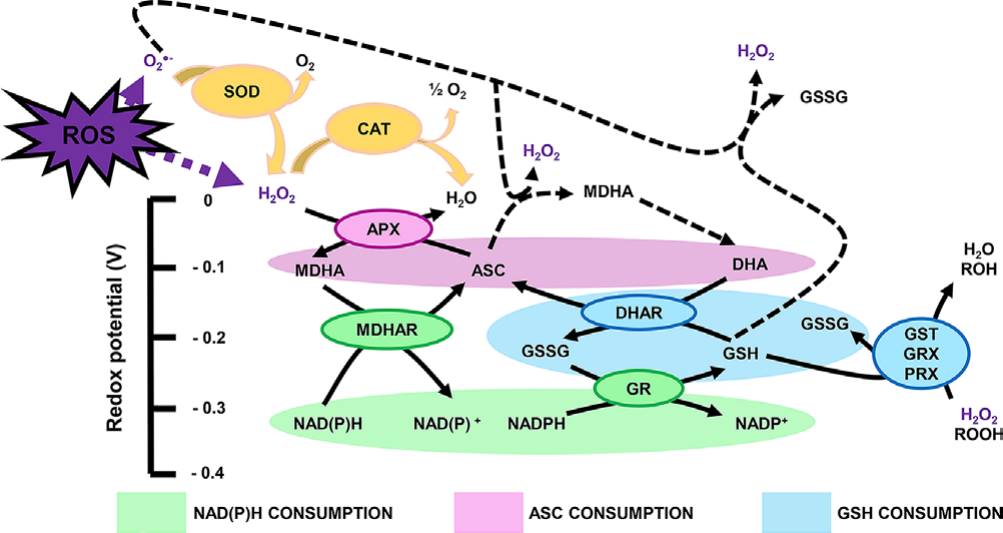
Major cellular redox buffers: a *ménage-à-trois* to process ROS. Plain and dashed arrows represent enzymatic and nonenzymatic reactions, respectively. ASC, reduced ascorbate; APX, ascorbate peroxidase; CAT, catalase; DHA, dehydroascorbate; DHAR, dehydroascorbate reductase; GSH, reduced glutathione; GSSG, glutathione disulphide; GR, glutathione reductase; GRX, glutaredoxin; GST, glutathione S-transferase; MDHA, monodehydroascorbate; MDHAR, monodehydroascorbate reductase; PRX, GRX-dependent peroxiredoxin; ROH, organic compound with alcohol group; ROOH, organic compound with peroxide group; SOD, superoxide dismutase.

Reactive oxygen species produced in the plant cell can be scavenged, or processed, by highly efficient antioxidant systems. If this were not the case, ROS levels exceeding the requirement of metabolic processes would damage cellular structures and functions involving nucleic acids, proteins and lipids (Apel and Hirt, 2004; Muñoz and Munné-Bosch, 2018). Antioxidants include metabolites with antioxidant properties, which in fruit are profuse in their diversity and quantity and are found in all organelles. Besides metabolites, the antioxidant machinery is composed of a few major enzymes that rapidly process ROS, i.e. catalase (CAT; EC 1.11.1.21), SOD (EC 1.15.1.1), ascorbate peroxidase (APX; EC 1.11.1.11), monodehydroascorbate reductase (MDHAR; EC 1.6.5.4), dehydroascorbate reductase (DHAR; EC 1.8.5.1), glutathione S-transferase (GST; EC 2.5.1.18), glutathione peroxidase (**GPX**; EC 1.11.1.9), glutathione reductase (GR; EC 1.8.1.7) and guaiacol peroxidase (**GX**; EC 1.11.1.7). Hence, redox biology presents another level of ambiguity as enzymes such as peroxidase or dismutase can be considered as both ROS-generating and ROS-processing components (**Figure 2**). These enzymes tightly link to the pool of the redox buffers ascorbate, glutathione and pyridine nucleotides, which serve as reductants to recycle repeatedly glutathione and ascorbate *via* the so-called Foyer-Halliwell (or ascorbate-glutathione) cycle (Foyer and Noctor, 2011) (**Figure 2**). In addition, thioredoxins (TRXs) are widely distributed small proteins, which modulate the redox state of target proteins *via* transfer reactions of thiol-disulphide using NADP(H) as a cofactor (Geigenberger et al., 2017). These ROS-processing systems are also important for fruit metabolism, and they could link to developmental processes or responses to environmental changes, as we detail further below.

### Fruit Antioxidants

**Figure 3:**
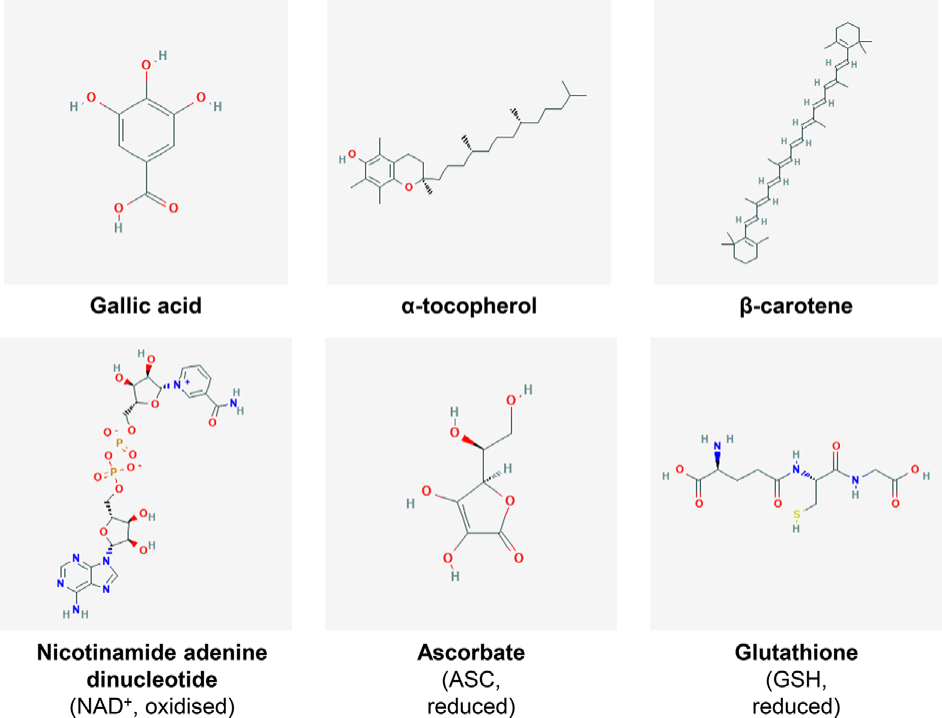
Examples of fruit antioxidants. Chemical structures of several metabolites presenting antioxidant properties. The major redox buffers NAD^+^, ASC and GSH are also presented. For further detail, refer to **Table 1**. Individual structures were obtained from PubChem (pubchem.ncbi.nlm.nih.gov/).

Fruits, especially citrus and berry fruits, are well-known sources of antioxidants conferring plenty of beneficial effects for human health (Gomes-Rochette et al., 2016). Because of their intricate oxidative metabolism (ROS production, described above), plants have developed a wide range of antioxidant metabolites as well as pathways to synthetize, catabolise and regenerate them. Basically, antioxidants refer to all biomolecules, including metabolites, which can process ROS and/or reactive nitrogen species to delay or avoid cell damage and for signalling processes (Nimse and Pal, 2015). Antioxidants can be distributed into several biochemical classes (**Figure 3**), including phenolics, terpenoids, thiol derivatives and vitamins, for which common metabolites and their antioxidant mechanisms are listed in **Table 1**. Terpenoids, also known as isoprenoids for their core structure, can be divided into several classes based on their carbon skeleton, and among them, carotenoids are the main group with more than 600 having been identified and characterised (Graßmann, 2005). They are pigments used for light harvesting, preventing photo-oxidation and increasing fruit attractiveness for seed dispersion (Young and Lowe, 2018). Carotenoids among other terpenoids are widely studied with respect to their antioxidant properties and biological effects in plants and mammals. Whilst antioxidants are often shared by plant species, most plant families have developed their own range of specific antioxidant metabolites within their botanical taxa. Quite importantly, some major redox buffers shared between species, such as ferredoxins, pyridine nucleotides, TRXs, glutathione and ascorbate, can be distinguished as they play a fundamental role in the development of plants and their responses to the environment and thus in plant performance (Balmer et al., 2004; Geigenberger and Fernie, 2014; Geigenberger et al., 2017; Noctor et al., 2018; Gakière et al., 2018a).

**Table 1:** Examples of major antioxidant metabolites present in fruits.

Biochemical class	Compound class	Antioxidative metabolite	Antioxidant activity	Effect on human health	Source example (per 100 g FW)	Key references
**Polyphenols**	Hydroxycinnamic acids	Caffeic acid	Scavenge ROS and peroxyl radicals Inhibit lipid peroxidation	Anti-inflammatory Preventive effects for diabetes Cardiovascular protective effects	0.1–1.3 mg in tomato 0.4–35 µg in blueberries	Fu et al., 2011 Wolfe et al., 2008 Wang et al., 2017 Olas, 2018
		Ferulic acid			0.2–0.5 mg in tomato 26–185 µg in blueberries	Martí et al., 2016 Wang et al., 2017
		p-coumaric acid			0–0.6 mg in tomato 89–225 µg in blueberries 15–42 mg in strawberries	Skupien and Oszmianski, 2004 Martí et al., 2016 Wang et al., 2017
	Hydroxybenzoic acid	**Gallic acid**	Scavenge peroxyl radicals and ROS		2–9 mg in different cultivars of blackberries	Wada and Ou, 2002 Wang et al., 2017
	Flavonoids	Anthocyanins	Scavenge free radicals Acylation of anthocyanins with phenolic acid increase the antioxidant activity Prevent lipid peroxidation	Neuroprotective effects Anti-cancer involved in treatment of cardiovascular diseases	154–1001 µg in blueberries of Cyanidin 25–40 mg in strawberries of total anthocyanins	Skupien and Oszmianski, 2004 Khoo et al., 2017 Wang et al., 2017 Olas, 2018
		Catechin	Prevent lipid peroxidation Scavenge NO and ROS	Regulate superoxide production Regulation of transcription factors involved in oxidative stress responses	180–338 µg in blueberies 6–19 mg in different cultivars of strawberries	Fraga et al., 2018 Wang et al., 2017 Skupien and Oszmianski, 2004
		Quercitin		Neuroprotective and cardioprotective effects Anti-cancer	0.7–4.4 mg in tomato 202–266 µg in blueberries	Chaudhary et al., 2018 Martí et al., 2016 Wang et al., 2017
	Stilbenes	Resveratrol	Scavenge ROS and peroxyl radicals Inhibit lipid peroxidation	Neuroprotective and cardioprotective effects	51–97 µg in blueberries	Wang et al., 2017 Cory et al., 2018
**Carotenoids**		Lycopene	Process singlet oxygen Trap peroxyl radicals Inhibit radical-induced lipid peroxidation Reduce ROS production by nonphotochemical quenching of chlorophyll fluorescence	Anti-inflammatory Pro-vitamin A activity, converted to retinoids after breaking (oculo protective effects) Enhance immune system Anti-proliferative and anti-carcinogenic	7.8–18.1 mg in tomato 1.82–3.6 g in different buffaloberry cultivars	Eldahshan and Singab, 2013 Martí et al., 2016 Chaudhary et al., 2018 Murillo et al., 2010 Graßmann, 2005 Riedl et al., 2013
		Zeaxanthin			200 µg in mandarins 7.92 mg in South American sapote 6 mg in orange pepper 340 µg in tomato	Murillo et al., 2010
		**β-Carotene**			0.1–1.2 mg in tomato 1.5–3.8 mg in apricot	Martí et al., 2016 Sass-Kiss et al., 2005
**Thiols**		**Glutathione**	Process ROS *via* enzymatic and non-enzymatic reactions ROS scavenging Maintain thiol equilibrium S-glutathionylation of Cys residues allowing regulation of central metabolism during oxidative stresses	Neuroprotective effects Involve in asthma prevention and treatment	1,3 mg in mango 210–298 µg in strawberries 16–19.5 mg in tomato	Ding et al., 2007 Fitzpatrick et al., 2012 Smeyne and Smeyne, 2013 Erkan et al., 2008 Martins et al., 2018 Noctor et al., 2018 Keutgen and Pawelzik, 2007
**Vitamins**	Tocochromanols	**α-Tocopherol (VE)**	Prevent lipid peroxidation by scavenging free radicals (donating hydrogens) using ascorbate to be regenerated Prevent the oxidation of carotenoids Essential macronutrient for human maintaining cell membrane integrity	Anti-anemia Neuroprotective effects	0.5–1,1 mg in tomato; 0.6–0.8 µg in MoneyMaker cultivar 1,6–3,2 mg in red sweet pepper 3.8 mg in green olives of total tocopherol + tocotrienols	Gugliandolo et al., 2017 Giovinazzo et al., 2004 Chaudhary et al., 2018 Dasgupta and Klein, 2014 Raiola et al., 2015 Chun et al., 2006 Knecht et al., 2015
		**Ascorbate (VC)**	Process ROS *via* enzymatic and non-enzymatic reactions Allow the regeneration of tocopherols and carotenoids	Anti-scurvy Anti-inflammatory Anti-cancer	10–15 mg in commercial cultivars of tomato and until 70 mg in ancestral cultivars 54–87 mg in different cultivars of strawberries 2.4–3g in camu-camu	Chaudhary et al., 2018 Martins et al., 2018 Stevens et al., 2007 Skupien and Oszmianski, 2004 Justi et al., 2000

Due to the wide diversity of fruit metabolites harbouring antioxidant activity, fruit antioxidants can process ROS in many ways. Most antioxidants spontaneously react with ROS, although enzymes such as APXs and glutaredoxins (GRX) catalyse several reactions. As previously mentioned, antioxidants remarkably participate in recycling pathways, such as the glutathione-ascorbate cycle, to maintain the redox state of the main redox buffers through the activity of GR, DHAR and MDHAR (**Figure 2**). The importance of such systems for fruit biology is detailed in Section 4.

### Three Major Cellular Redox Buffers: A Ménage-À-Trois to Manage ROS

Ascorbate (ASC) and glutathione (GSH) sit at the top of plant soluble antioxidants because they process ROS rapidly using specific enzymes such as peroxidases belonging to the ascorbate-glutathione pathway (Foyer and Noctor, 2011) (**Figure 2**). In brief, ROS react preferentially with GSH and ASC: the latter can reduce H_2_O_2_*via* APX to produce water and MDHA that will be reduced by MDHAR using NAD(P)H, or be transformed spontaneously in DHA that will be reduced by DHAR using GSH (**Figure 2**). These repetitive redox cycles allow for the regeneration of the pools and the maintenance of the cellular redox buffers in a highly reduced state in most cellular compartments under unstressed conditions. In addition, pyridine nucleotides (i.e. NAD(P)H and NAD(P)^+^; **Figure 3**) are crucial for the regeneration of GSH and ASC through GR and MDHAR enzymes as well as being involved in other metabolic pathways, thereby linking redox homeostasis to central metabolism (Gakière et al., 2018a). Strikingly, fruit-specific concentrations and redox states of the pools are difficult to find in the literature (**Table 2**). In unstressed conditions, ASC and GSH are in a highly reduced state (> 90%), NAD(H) is 60% to 65% reduced, and NADP(H) is at 90% reduced in red ripe tomato fruits (Araújo et al., 2012; Centeno et al., 2011; Jimenez et al., 2002). However, NAD(H) is 12% to 20% reduced, and NADP(H) is 50% to 55% reduced in orange, apple, pear and grapefruits (Bruemmer, 1969), which is congruent with the redox status of photosynthetic tissues (Gakière et al., 2018b). This clearly suggests a diversity in fruit redox homeostasis as fruit growth influences the redox state of pyridine nucleotides. Furthermore, these three major cellular redox buffers display distinct redox potential: −0.1, −0.23 and −0.32 mV for the ASC/DHA, GSH/GSSG and NAD(P)^+^/NAD(P)H couples, respectively (**Figure 2**). In this case, as pyridine nucleotides have a lower redox potential, they will be detrimental for electron transfer to GSH and ASC during redox mechanisms.

**Table 2:** Examples of ASC, GSH and NAD/P(H) sources in fruits.

	Source example (per 100 g FW)	References
**ASC**	10 to 15 mg in tomato 54–87 mg in strawberries 2.4-3 g in camu-camu	Stevens et al., 2007 Skupien and Oszmianski, 2004 Justi et al., 2000
**GSH**	1.3 mg in mango 16–19.5 mg in tomato 210–298 µg in strawberries	Ding et al., 2007 Giovinazzo et al., 2004 Cervilla et al., 2007 Keutgen and Pawelzik, 2007
**NAD^+^**	3.21 mg in red fruits and 2.22 mg at breaker stage in tomato 780 µg in orange 400 µg in grapefruit	Osorio et al., 2013b Centeno et al., 2011 Bruemmer, 1969
**NADH**	5.82 mg in red fruits and 4.94 mg at breaker stage in tomato 170 µg in orange 50 µg in grapefruit	Osorio et al., 2013b Centeno et al., 2011 Bruemmer, 1969
**NADP^+^**	0.46 mg in red fruits and 0.77 mg at breaker stage in tomato 89 µg in orange 69 µg in grapefruit	Osorio et al., 2013b Centeno et al., 2011 Bruemmer, 1969
**NADPH**	3.88 mg in red fruits and 3.23 mg at breaker stage in tomato 119 µg in orange 89 µg in grapefruit	Osorio et al., 2013b Centeno et al., 2011 Bruemmer, 1969

## The Importance of the Redox Hub for Fruit Signalling

**Figure 4:**
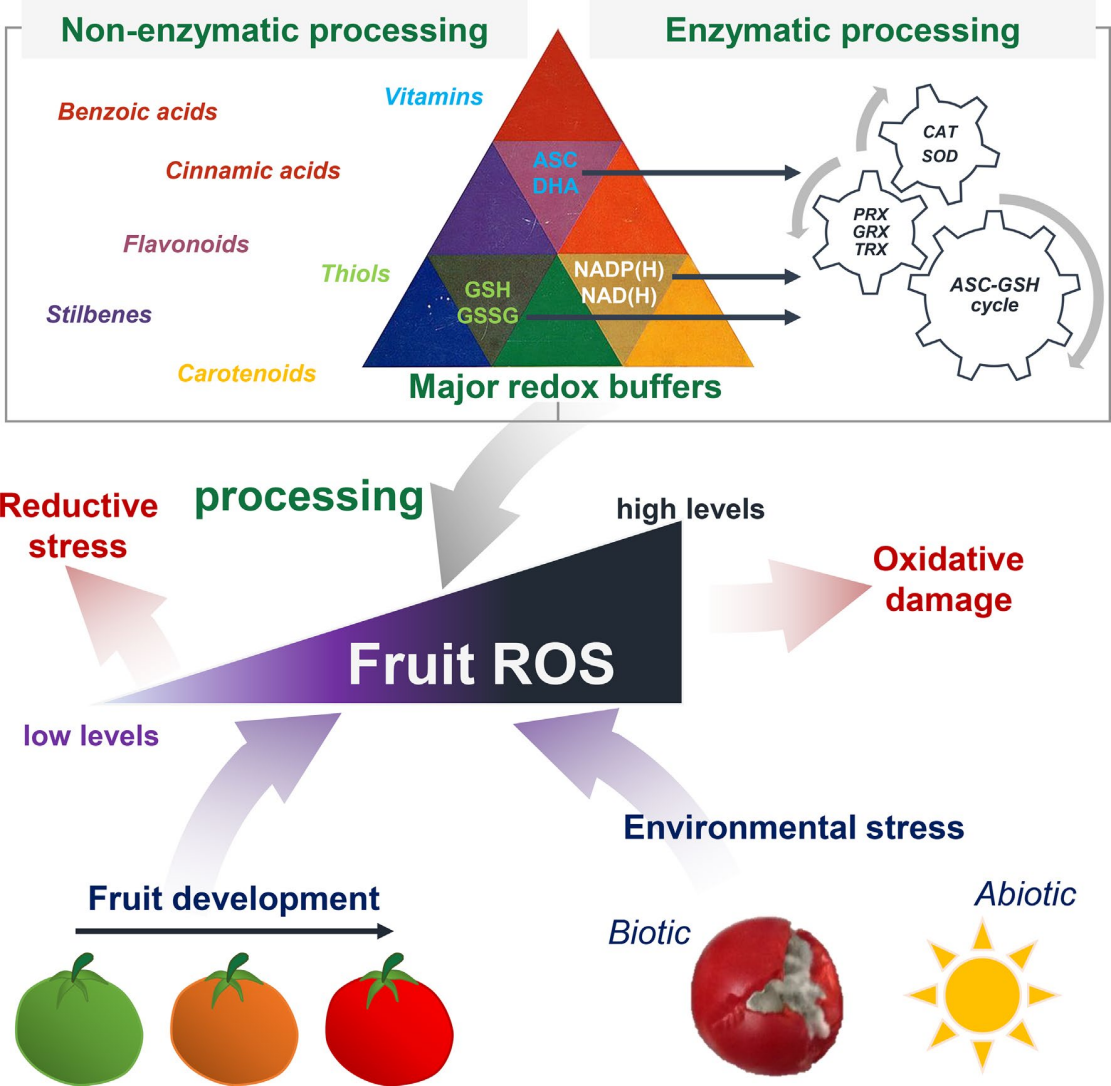
Pivotal role of redox signalling for fruit growth and stress responses. Fruits produce ROS and other redox signals during development and in response to environmental stress. Reactive oxygen species homeostasis is finely tuned between ROS production and processing. This involves several enzymatic and non-enzymatic mechanisms, including antioxidant metabolites and major redox buffers (NAD/P(H), ASC and GSH) that allow antioxidant cycles to process excess of ROS. When ROS levels are too high, the resulting oxidative stress would damage cellular structures.

The redox hub consists of all the molecular partners able to generate, process or trigger oxidative signals, whilst the resulting redox signalling can modulate the physiology of plant organs including fruits (Mittler, 2017; Noctor et al., 2018). Fruits are a major source of central metabolites (Osorio et al., 2013a; Roch et al., 2019), such as carbohydrates, lipids, amino and organic acids, but also vitamins and other antioxidant metabolites that play important roles in fruit biology (**Figure 3**). Besides, redox status is also at the heart of the control of metabolic processes (Geigenberger and Fernie, 2014). One among many reasons is the prominence of pyridine nucleotides (NAD/P(H)) as master regulators of hundreds of biochemical reactions (Gakière et al., 2018a), together with ascorbate/dehydroascorbate (ASC/DHA) and glutathione (GSH/GSSG) couples (Noctor et al., 2018). In this context, we will present recent advances in our understanding of key spatiotemporal redox signals that occur during developmental processes and in response to environmental changes, including redox buffers that balance the redox poise (**Figure 4**).

### Redox and Central Metabolism During Fruit Development

Fruit development comprises three main phases: cell division, cell expansion and ripening. As green organs, young fruits and leaves share some similarities due to the presence of photosynthetically active chloroplasts driving central metabolism, hence developmental processes (Cocaliadis et al., 2014). Fruit photosynthesis can contribute to the production of starch, which can then be turned into soluble carbohydrates during ripening. In tomato, a number of studies points towards the importance of the mitochondrial malate valve in transmitting redox status to the plastids, which will influence plastidial metabolism (Centeno et al., 2011; Osorio et al., 2013b). In fact, decreasing malate content in the growing fruit could stimulate the activation state of AGPase (EC 2.7.7.27), leading to increased starch and soluble sugar pools in ripening tomato. Intriguingly, such metabolic repercussions tend to provide tolerance of tomato to water loss, wrinkling and pathogenic infections. This supports the paradigm of a versatile role of redox signals in metabolic regulation throughout development and in response to stress (Tian et al., 2013). Another hallmark of this growth phase is when chloroplasts become chromoplasts by losing green chlorophylls at the expense of coloured antioxidants like carotenoids (Lado et al., 2015; Martí et al., 2009). Concomitantly, the expression of nuclear- and plastid-encoded photosynthetic genes drops as the fruit ripens. It is noteworthy that ROS synthesis influences the accumulation of carotenoids (Pan et al., 2009), which are major scavengers of singlet oxygen, specifically β-carotene, tocopherol and plastoquinone (Miret and Munné-Bosch, 2015). Besides carotenoids, anthocyanins can accumulate in the growing fruits and contribute to both red/purple/blue colours and antioxidant properties [**Table 1**; (Muñoz and Munné-Bosch, 2018)]. Whilst carotenoids accumulate primarily within lipophilic membranes, anthocyanins are stored in the vacuole where their colour depends on their chemical structure, which is influenced by vacuolar pH (Jaakola, 2013). In this context, recent studies have reported a critical role for epigenetic processes in growing tomato fruit by linking DNA demethylation levels with transcriptomic changes of genes involved in fruit antioxidant biosynthesis (e.g. flavonoids, carotenoids) (Lang et al., 2017). Conversely, however, development and ripening of orange, a nonclimacteric fruit, were correlated with an increase in DNA methylation levels, together with repression of photosynthetic genes (Huang et al., 2019). Thus, fruit development is likely to present remarkable discrepancies in terms of redox signals, their source (e.g. chloroplastic, mitochondrial, peroxisomal, apoplastic) and the duration and extent of oxidative stress, even at early stages of fruit growth in comparison to leaves (Muñoz and Munné-Bosch, 2018).

Ripening is an important end process of fruit development that involves multiple molecular regulations (Osorio et al., 2013a). It is mediated by redox signalling, more specifically during the chloroplast-to-chromoplast transition and in the mitochondrial compartment, where protein carbonylation occurs and respiration rates increase, thus affecting the redox state when sugar supply becomes limiting (Qin et al., 2009a; Tian et al., 2013). As the fruit ripens, oxidative stress progressively augments, like in peach, tomato, pepper and grape berries (*Vitis vinifera*), where H_2_O_2_ pools accumulate upon changes in skin colour (Jimenez et al., 2002; Martí et al., 2009; Qin et al., 2009a; Pilati et al., 2014; Kumar et al., 2016). In fact, it is assumed that ROS accumulation produces two distinct peaks during fruit growth: first at the onset of ripening and second at overripening either preharvest or postharvest (Muñoz and Munné-Bosch, 2018). It is possible that increased oxidative stress might favour fruit softening, which is beneficial for seed release (Jimenez et al., 2002). This would explain why short life tomato cultivars are redox-stressed and present lower antioxidant activities (Cocaliadis et al., 2014).

Because of ROS imbalance, oxidative signals need processing *via* cellular redox buffering and the antioxidant machinery to avoid cellular damage (**Figure 4**). In ripening grape berries, accumulated levels of H_2_O_2_ are accompanied with a concomitant stimulation of CAT activity (Pilati et al., 2014). Moreover, under oxidative stress, increases in activities of APX, MDHAR and GR are seen in peach (Camejo et al., 2010). Contrasting observations are reported for peroxidases: ripening phase is associated with increased PX activities in mango, apples and banana fruit, whilst tomato, strawberry and capsicum show a decline in these activities (Pandey et al., 2012, and references therein). The importance of antioxidant systems for ripening is also exemplified in grapevines where there is a strong developmental modulation of ASC metabolism at the biosynthetic, recycling and catabolic levels (Melino et al., 2009). Fruit growth of wine grapes can witness a gradual induction of ASC biosynthesis and subsequent changes in the accumulation of ASC and two derivatives: tartaric and oxalic acids. Whilst immature berries showed a swift accumulation of ASC together with a low ASC/DHA redox ratio, ripe fruits instead showed an increased accumulation of ASC and higher ASC/DHA ratio. Additionally, acerola, an exotic fruit cultivated mostly for its ascorbic acid content, shows differential regulation of MDHAR and DHAR genes during fruit ripening (Eltelib et al., 2011). A comparison of ASC metabolism in mandarin and orange, two citrus species harbouring different ASC contents in pulp, deepens our understanding of the differences in ASC concentrations in fruit (Yang et al., 2011b). This study revealed that higher ASC in ripening orange was associated with an augmented expression of four genes involved in ASC biosynthesis, encoding GDP-d-mannose-3′,5′-epimerase (EC 5.1.3.18), GDP-l-galactose-phosphorylase (EC 2.7.7.69), l-galactose dehydrogenase (EC 1.1.1.316) and l-galactono-1,4-lactone dehydrogenase (EC 1.3.2.3), together with attenuated activities of ASC oxidase and ASC peroxidase, which are involved in ASC degradation. Another elegant work on isolated mitochondria from ripening tomato fruits has reported a global stimulation of the ASC-GSH cycle at the enzyme level (López-Vidal et al., 2016). Recently, a system biology approach in tomato has been conducted based on large-scale transcriptomic, proteomic, metabolic and phenotypic data for orange fruit of RNAi lines for three enzymes involved in ASC metabolism (AO, GLD and MDHAR) (Stevens et al., 2018). The ASC redox state has been reported to influence the expression of genes involved in cellular protein synthesis and stability and ribosomal function. Besides redox functions, synthesis of ASC is also crucial for tomato fruit growth (Garcia et al., 2009; Mounet-Gilbert et al., 2016), as exemplified by profound growth stunting of tomato fruits silenced in mitochondrial ASC synthesis (Alhagdow et al., 2007). Another recent study of protein turnover at global scale in the developing tomato fruit revealed a stage-specific response of protein profiles that were associated to various redox functions (Belouah et al., 2019). Changes in redox-related proteins were represented in the young fruit (e.g. SOD, APX) and at ripening (e.g. MDHAR, GR). Hence, ASC metabolism appears to be central to redox homeostasis during fruit development.

Upon stress and senescence (i.e. ageing), oxidative alterations can drastically target proteins, resulting in conformational changes and thus impairing their catalytic functions. Methionine (Met) and cysteine, which contain sulphur, are probably the most susceptible to ROS oxidation (Davies, 2005). In the case of Met, oxidation can be reversed by Met sulphoxide reductase (MSR, EC 1.8.4.11/12) (Emes, 2009; Rey and Tarrago, 2018), which has been reported to play a role in senescing litchi fruit through down-regulation of *MSR* genes (Jiang et al., 2018). In leaves, previous works have suggested a link between MSR and the homeostasis and redox balance of NAD/P(H) (Pétriacq et al., 2012, Pétriacq et al., 2013). Besides MSR, a stimulation of the antioxidant systems in tomato fruit mitochondria has been reported to be associated with a differential carbonylation of mitochondrial proteins in breaker and light red tomato fruits, which might participate in protein degradation and cellular signalling (López-Vidal et al., 2016). Besides targeting proteins, aging of fruit encompasses other redox-related changes. In the pulp of Kyoho grape, postharvest senescence and rotting are accompanied by an accumulation of oxidative signals (e.g. malondialdehyde, hydrogen peroxide, superoxide anion) and a concomitant depletion of several antioxidant systems (e.g. ascorbate, flavonoids, total phenolics, reducing sugars) (Ni et al., 2016). Interestingly, exogenous treatment with hydrogen sulphide could alleviate those redox perturbations by enhancing the activity of antioxidant enzymes, such as CAT and APX, and by attenuating those of lipoxygenase in the pulp and peel of Kyoho grape.

Additionally, not only central metabolism links to redox signalling in fruit but also more specialised pathways involving phytohormones (Symons et al., 2012; Leng et al., 2013). In red raspberry, a nonclimacteric crop fruit, the stage of ripeness at the time of harvest determines the antioxidant contents (e.g. anthocyanins, ellagitannins, vitamins C and E, carotenoids) (Beekwilder et al., 2005; Miret and Munné-Bosch, 2016). Application of the carotenoid-derivative hormone ABA after fruit set modulates the ASC/DHA ratio in young berries and more than doubles ASC pools in ripe fruit. Such an effect was partially explained by alterations of ASC oxidation and recycling through the activities of AO, APX, DHAR and MDHAR (Miret and Munné-Bosch, 2016). In postharvest conditions, fruit decay is a major issue caused by perturbation of the redox balance, including ROS production (Pétriacq et al., 2018). Thus, antioxidant mechanisms (e.g. ASC total pool and redox state, ASC-GSH cycle) are important actors throughout fruit growth, which is further evidence for the idea that ROS act as metabolic by-products requiring a finely tuned homeostasis (**Figure 4**). In an agri-food context, further research is required to disentangle the implication of each redox event occurring during fruit development, so that efficient strategies can be adopted to improve fruit production and storage.

Nevertheless, the active depiction of redox fluxes by deciphering redox signatures in plant biology is extremely tedious, if not impossible, probably due to the extreme reactivity of ROS and related redox signals and to the intricacy of the redox hub. However, a very interesting and promising alternative to measurements of redox pools and antioxidant systems is the use of mathematical modelling of metabolism, in particular for redox branches. In the context of central metabolism, previous studies elegantly shed a different light on climacteric respiration in tomato fruit using stoichiometric models (Colombié et al., 2015, Colombié et al., 2017). Using a medium‐scale stoichiometric model, energy and the redox cofactors NAD(H) and NADP(H) were defined as internal metabolites and balanced so that constraining of the metabolic network was possible not only through C and N homeostasis, but also through the redox and energy status (Colombié et al., 2015). This model suggested a consistent requirement of NADPH for biomass synthesis and demonstrated that higher ATP hydrolysis was required for growth starting at the end of cell expansion and that a peak of CO_2_ was released at the end of tomato ripening. This coincided with climacteric respiration of tomato fruit and involved energy dissipation by the AOX (**Figure 1B**), a redox marker of the mitochondrial compartment (Polidoros et al., 2009; Pétriacq et al., 2016). This was further confirmed by a more detailed stoichiometric model of the respiratory pathway, including AOX and uncoupling proteins (Colombié et al., 2017). Moreover, the recent flux analysis performed with grape cells under nitrogen limitation showed differently regulated fluxes were involved in the flavonoid (phenylpropanoid) pathway and in major carbon fluxes supporting a strong link between central metabolism and cell redox status by energy (ATP) and reducing power equivalents (NADPH and NADH) (Soubeyrand et al., 2018). Thus, mitochondrial function plays a notable role along fruit development in mitigating the redox poise upon an imbalance between energy supply and demand. In complement, when omics strategies failed to measure oxidative fluxes accurately, kinetic modelling of metabolism has proven to be a complementary and promising approach as it offers, with enzymatic and metabolic parameters, the possibility to describe quantitatively fluxes of cycling pathways such as redox metabolism. For instance, this was achieved previously for sucrose metabolism in the developing tomato fruit *via* a model of 13 differential equations describing the variations of hexoses, hexoses-phosphates and sucrose as a function of 24 enzyme reactions (Beauvoit et al., 2014). Similar approaches to redox cycles are necessary to obtain novel insights into the active redox dynamics involved in fruit biology.

### The Key Role of ROS and Cognate Redox Signals in Fruit Responses to Environmental Constraints

The generation of ROS is a crucial process in response of plants to a changing environment and contributes to establish adaptive signalling pathways (Noctor et al., 2014). Oxidative stress typically comes as a secondary stress after primary stresses, whether they are abiotic constraints (**Figure 4**), such as drought or flooding, wounding, high light, cold or heat stress or biotic stresses including pest attacks or bacterial and fungal infections. Fruits are no exception to this rule: ROS can originate from NADPH oxidases (**Figure 1B**), specifically with respect to biotic and abiotic environmental challenges (Torres and Dangl, 2005; Suzuki et al., 2011; Mittler, 2017). Upon cold stress in apple fruits, NADPH oxidases might function *via* a regulatory node that integrates ethylene and ROS signalling pathways (Zermiani et al., 2015). In strawberry fruits, recent identification of NAPDH oxidase genes indicated that *FvRbohA* and *FvRbohD* might be involved in cold stress and defence responses (Zhang et al., 2018).

At present, it is assumed that major redox couples (NAD/P(H), ASC, GSH) are integral regulators of stress responses in plants (**Figure 4**), including both abiotic and biotic stresses (Pétriacq et al., 2013; Noctor and Mhamdi, 2014; Smirnoff, 2018; Gakière et al., 2018a). For instance, exogenous application of NAD^+^ confers resistance to citrus canker disease in citrus (Alferez et al., 2018). In coherence with a modulation of these redox buffers, the antioxidant system further contributes in processing excess of ROS within stressed tissues (Foyer and Noctor, 2011; Smirnoff and Arnaud, 2019) (**Figure 4**). Additionally, redox processes dominate hormonal signalling *via* the stress hormones salicylic (SA), jasmonic (JA) and abscisic acids, which play a critical role in metabolic adjustments under stress conditions (Leng et al., 2013; Geigenberger and Fernie, 2014; Gakière et al., 2018a). Thus, a complex signalling network is devoted to shaping the fruit responses to stress. However, the interrelation between these multiple signalling partners is poorly understood, and its study will necessitate further research.

As for developmental processes (**Figure 4**), a hallmark of plant responses to stress is the activation of the ASC-GSH cycle (**Figure 2**). Upon arsenic and silicon exposure, fruits of two tomato cultivars exhibited different but profound redox perturbations of H_2_O_2_ and antioxidant contents (e.g. lycopene, carotenoids and phenolics), ASC and GSH redox states and lipid peroxidation (Marmiroli et al., 2017a). Alternatively, a detailed proteomic study on tomato fruit confirmed the implication of ASC- and GSH-related proteins in response to this abiotic stress (Marmiroli et al., 2017b). Some of these redox alterations (H_2_O_2_, ASC and GSH redox states, total carotenoids and phenolics) were proposed as reliable arsenic exposition biomarkers for further studies that could broaden our knowledge on arsenic-induced abiotic stress in fruit (Marmiroli et al., 2017a). Besides arsenic, hot air treatment of strawberry fruits directly triggered the induction of antioxidant enzymes (e.g. CAT, APX and SOD), which further leads to a reduction of necrotrophic lesions caused by the fungal pathogen *Botrytis cinerea* (Jin et al., 2016). Additionally, a study of cold and light stress in tomato fruit unveils an interaction between temperature and light to modulate synthesis, recycling and oxidation of ASC in fruit (Massot et al., 2013). Light promoted the accumulation of ASC and GSH in tomato fruit, thus supporting the hypothesis of a stimulation in ASC synthesis by light (Gautier et al., 2009; Massot et al., 2012; Baldet et al., 2013; Smirnoff, 2018).

Redox signalling is associated with physiological disorders in fruits stored under multiple environmental stresses, such as for pome fruit, where redox-related metabolites are likely to accumulate (e.g. γ-aminobutyrate [GABA]) or rapidly decline (e.g. ASC, GSH) after exposure to low O_2_ and/or elevated CO_2_ environments (Lum et al., 2016). This in turn results in disturbances of the energetic and oxidative balance. In this context, both GABA and antioxidant metabolism are regulated by NAD/P(H) ratios, which confirms the tight link between cellular redox buffers and the regulation of oxidative metabolism (Trobacher et al., 2013; Lum et al., 2016) (**Figure 4**). A characterisation of TRX genes in harvested banana fruit suggests that the protein MaTrx12 regulates redox homeostasis, which impacts chilling tolerance (Wu et al., 2016). In tomato fruit, a combination of deep sequencing and bioinformatics revealed 163 circular RNAs that exhibited chilling responsive expression, among them several ones predicted to be involved in redox reactions and various stress signalling pathways (e.g. heat/cold shock protein, energy metabolism, hormonal responses, salt stress, cold-responsive transcription factors) (Zuo et al., 2016).

Infection of fruits with pathogenic microbes is a pressing issue due to dramatic postharvest diseases that can claim up to 50% of the total production worldwide (Romanazzi et al., 2016; Pétriacq et al., 2018). Resistance inducers have been used as promising strategies to elicit fruit defences against phytopathogens (Pétriacq et al., 2018). A global transcriptional analysis of strawberry fruit has demonstrated that the fungal elicitor chitosan and the salicylate-mimicking compound benzothiadiazole modulate chloroplastic signals to trigger various defence responses through redox alterations (e.g. *PX*, *GST*, *GRX*) (Landi et al., 2017). Accordingly, induction of sweet orange with chitosan or salicylic acid also alters the redox status of the cell (e.g. TRX, SOD, PX), as exemplified through RNAseq data (Coqueiro et al., 2015). Another example comes from *Peronophythora litchii–*infected litchi fruits that exhibit lower infection symptoms after treatment with a novel chitosan formulation (Jiang et al., 2018). Disease tolerance was correlated in litchi pericarp with higher activities of defensive (e.g. chitinase, phenylalanine lyase, glucanase) and antioxidant enzymes (e.g. SOD, CAT, APX), a lower O_2_^−^ generation rate and lower malondialdehyde levels and higher contents of redox buffers including ascorbic acid and glutathione and reducing power. Moreover, priming of tomato seedlings with β-aminobutyrate (BABA), a novel phytohormone (Thevenet et al., 2017), confers resistance of tomato fruits to the fungal pathogen *B. cinerea* through metabolic rearrangements including antioxidant (e.g. flavonoids, polyphenols) and ABA contents (Wilkinson et al., 2018). This resistance was also associated with a delay in fruit ripening, which suggests a metabolic trade-off for defence metabolism versus fruit growth. Together, phytopathologic studies confirm the trigger of an oxidative burst in infected fruit tissues, for which excess ROS are mitigated both by a stimulation of enzymatic antioxidant systems and nonenzymatic protective, scavenging molecules (Tian et al., 2013). Hence, unsurprisingly, induction of antioxidant functions has proven to be effective in controlling postharvest diseases in fruits (Romanazzi et al., 2016; Pétriacq et al., 2018).

### Practical Applications Towards Modifying Redox Metabolism in Fruits

Although the precise functions of redox regulators remain to be evidenced, a few practical applications are currently explored towards modifying redox biology in fruits. From a human health perspective, fruit redox metabolism received much attention since fruits and vegetables are major sources of essential antioxidative metabolites and thus recommended in human diet (e.g. five a day, http://www.fao.org/). Due to the intensively studied health effects of antioxidants for their numerous benefits for aging, cancer and chronic disorders, research focused on strategies to increase the antioxidant contents in consumable product. Moderate success has been obtained in engineering plants to increase antioxidants content such as ASC, GSH and vitamin E (Wargovich et al., 2012; Gallie, 2013). However, the *Golden Rice*, enriched in β-carotene (provitamin A), remains a successful story for redox application in crops combining plant biotechnologies, antioxidant synthesis pathway and human health (Botella‐Pavía and Rodríguez‐Concepción, 2006). Nevertheless, due to the importance of ROS signalling in developmental processes, the modulation of oxidative mechanisms can alter fruit growth. For instance, engineering tomato fruits to increase levels of antioxidants by enhancing chloroplast functions results in longer-lasting and firmer fruits (Mehta et al., 2002; Zhang et al., 2013). Thus, future applications need to consider the spatial and temporal regulations of redox homeostasis during plant development to improve significantly plant productivity.

Fruit physiological disorders during storage under multiple environmental stresses are also associated with redox perturbations (Lum et al., 2016). Fruit decay is a major issue caused by changes of the redox balance, including ROS production, in postharvest conditions (Pétriacq et al., 2018). From an agri-food perspective, chilling stress is oxidative but also particularly critical as low temperatures are often used to delay senescence of many fruits (Lallu, 1997; Bustamante et al., 2016; Valenzuela et al., 2017; Alhassan et al., 2019). Reactive oxygen species accumulate during fruit overripening, which thus puts the improvement of fruit storage conditions in the forefront of redox signalling applications (Muñoz and Munné-Bosch, 2018). Furthermore, diverse chemical treatments have been identified to limit ROS accumulation by affecting either their production or processing. For instance, nitric oxide postharvest treatment in cucumber was associated with a decrease in ROS content and an increase of APX, CAT and SOD activities (Yang et al., 2011a; Liu et al., 2016). Other examples come from the use of chlorine dioxide fumigation in longan fruit that displays a reduction in enzymatic fruit browning (Saengnil et al., 2014) and ozone applications in citrus industry that allow to improve fruit shelf-life (Karaca, 2010). In addition, the plant defence hormones methyl-jasmonate (MeJA) and methyl-salicylate (MeSA) promote AOX gene expression in green pepper (Purvis, 1997). More recently, it was reported that MeJA also improved chilling tolerance of cucumber by increasing both CAT gene expression and enzyme activity (Liu et al., 2016). Biotechnological approaches have been further used to reduce oxidative stress in fruits mostly by overexpressing main ROS-processing enzymes (**Figure 2**) but also by increasing the total antioxidant content. In this context, anthocyanin- and flavonoids-enriched mango fruits have shown a better tolerance to cold during storage (Sudheeran et al., 2018).

Importantly, practical applications to modulate redox metabolism trigger plant resistance to biotic stresses. Fruits can suffer substantial yield losses from diseases as fruit decay at a postharvest level can claim up to 50% of the total production worldwide (Pétriacq et al., 2018). Given that ROS signalling is central to plant-pathogen interactions (Mittler, 2017), and main redox buffers are linked to defence hormonal signalling (Pétriacq et al., 2013; Pétriacq et al., 2016; Pétriacq et al., 2018), diverse treatment building on hormonal and redox signalling has shown a lower disease incidence and symptoms. For instance, nitric oxide treatment inhibits anthracnose (*Colletotrichum gloeosporioides*) in ripening mango (Hu et al., 2014) and further improves chilling tolerance in banana fruit *via* an induction of the antioxidative defence system (Wu et al., 2014). Additionally, MeSA and MeJA treatments can be used to stimulate pathogen resistance and increase the antioxidant content without affecting fruit quality in kiwi, tomato and peach (Tzortzakis and Economakis, 2007; Zhang et al., 2008; Fatemi et al., 2013).

## Concluding Remarks and Future Outlooks

Not before time, the simple Manichean belief of ‘good’ reductants and ‘bad’ oxidants, such as ROS, has become erroneous. There is so much to learn from future molecular studies of redox metabolism, particularly in fruit, for which an obvious lack of fundamental knowledge needs to be addressed. Reactive oxygen species production and cognate redox signals are key to harmonious metabolism and contribute to establishing adaptive signalling pathways throughout development and in response of fruits to environmental events. Whilst redox buffers, specifically ascorbate, clearly appear at the forefront of oxidative regulation, these redox mechanisms also seem to depend on the fruit species. Recent years have witnessed a growing interest in developing both analytical technologies (e.g. LCMS, NMR, ROS detection, redox proteomics) and mathematical modelling to provide quantitative description of the central metabolism and specialised pathways including antioxidant processes (Qin et al., 2009a; Beauvoit et al., 2014; Colombié et al., 2015; Colombié et al., 2017; Deborde et al., 2017). In tomato fruit, for instance, spatially resolved distribution of metabolites including antioxidants will help to decipher the involvement of such redox compounds in physiological responses (Nakamura et al., 2017).

Studying key spatiotemporal redox processes involved in fruit is of paramount importance. Numerous fruits, such as the ones from the Solanaceae family (e.g. tomato, pepper, eggplant), not only contain a cocktail of antioxidants (vitamins A and C, flavonoids), but also domestication of these plants has reduced the content in prohealth molecules such as vitamin C. Indeed, ascorbate was higher in ancestral cultivars of tomato (Gest et al., 2013b; Palma et al., 2015). These are among the many reasons for ascorbate to be at the heart of research on the plant redox hub, where plant scientists endeavour to increase fruit ASC content, which should improve human nutrition and plant tolerance to stress (Macknight et al., 2017). Progress in understanding the molecular signatures involved in the redox regulations that link central metabolism and stress pathways will help to define novel strategies for optimal fruit production and storage (Beauvoit et al., 2018).

## Author Contributions

All authors contributed to writing this review.

## Funding

The authors are also grateful to the MetaboHUB (ANR-11-INBS-0010) and PHENOME (ANR-11-INBS-0012) projects for financial support. The doctoral school Sciences de la Vie et Santé at Université de Bordeaux is also acknowledged for granting PP with PhD funding for GD (bourse fléchée ministérielle 2018-2021).

## Conflict of Interest Statement

The authors declare that the research was conducted in the absence of any commercial or financial relationships that could be construed as a potential conflict of interest.

## Abbreviations

ABA, Abscisic acid; AGPase, ADP-glucose pyrophosphorylase; AltDH, Alternative dehydrogenases; AO, Ascorbate oxidase; AOX, Alternative oxidase; APX, Ascorbate peroxidase; ASC, Ascorbate; BABA, β-Aminobutyrate; CAT, Catalase; Chl, Chlorophyll; DHA, Dehydroascorbate; DHAR, Dehydroascorbate reductase; GABA, γ-Aminobutyrate; GP, Guaiacol peroxidase; GPX, Glutathione peroxidase; GR, Glutathione reductase; GRX, Glutaredoxins; GSH, Glutathione (reduced form); GSSG, Disulphide glutathione (oxidised form); GST, Glutathione S-transferase; JA, Jasmonic acid; LCMS, Liquid chromatography–mass spectrometry; MDHA, Monodehydroascorbate; MDHAR: Monodehydroascorbate reductase; Met, Methionine; MSR, Met sulphoxide reductase; NAD, Nicotinamide adenine dinucleotide; NADP, Nicotinamide adenine dinucleotide phosphate; NMR, Nuclear magnetic resonance; PS, Photosystem; PX, Peroxidase; RBOH, Respiratory burst oxidase homolog; ROS, Reactive oxygen species; ^1^O_2_, Singlet oxygen; H_2_O_2_, Hydrogen peroxide; O_2_^•−^, Superoxide anion; OH•, Hydroxyl radical; SA, Salicylic acid; SOD, Superoxide dismutase; TCA, Tricarboxylic acid; TRXs, Thioredoxins; XDH, Xanthine dehydrogenases.
